# Factors associated with the perceived need for assistance from voluntary services in home-based older adults in Chinese urban areas: a cross-sectional study

**DOI:** 10.1186/s12877-023-04354-7

**Published:** 2023-10-06

**Authors:** Lei Huang, Hongyan Wu, Fengjian Zhang, Huimin Zhao, Yuqin Chen, Mingjiao Feng, Yanjie You, Xiao Peng, Chunyan Guan, Yilan Liu

**Affiliations:** 1 Department of Nursing, Union Hospital, Tongji Medical College, Huazhong University of Science and Technology, Wuhan, 430022 China; 2 School of Nursing, Tongji Medical College, Huazhong University of Science and Technology, Wuhan, 430030 China

**Keywords:** Older adults, Assistance needs, Voluntary services, Volunteer, Home-based

## Abstract

**Background:**

With China's rapidly aging population, meeting the diverse care needs of senior citizens is becoming more challenging. Although voluntary social services have numerous advantages and are popular among older adults, there is little information on the need for assistance from volunteer-based social services, particularly those with a medical background, and influencing factors among urban home-based older adults. This study aimed to assess the need for assistance from voluntary services and related factors among urban home-based older adults in China.

**Methods:**

A cross-sectional study was conducted in 2022 on communities in four cities in China. The 27-item Home-Based Older Adults Assistance Need Scale was used to measure the assistance needs of 498 participants aged 60 and above. Multiple linear regression models were conducted to identify salient variables associated with the need for assistance from voluntary services.

**Results:**

The mean score of the need for assistance from voluntary services was 88.60 ± 24.37. The mean scores of the items examining four dimensions, namely, health maintenance, visiting communication, social intercourse, and daily life, were 3.64 ± 1.08, 3.49 ± 1.04, 3.33 ± 1.08, and 2.78 ± 1.08, respectively. The level of depression, willingness to assist older adults, attaching importance to health preservation, ability to self-comfort, desire to accept assistance from others, and the presence of more children or none at all were all positively correlated with the perceived need for assistance from voluntary services. In contrast, social care obtained from visiting medical institutions was negatively correlated. These seven factors explained 28.5% of the total variance.

**Conclusions:**

Urban home-based older adults in China were found to have significant requirements for assistance from volunteer services, and several complex factors were associated with more significant assistance needs. These findings may encourage the extremely limited numbers of social volunteers, particularly those with a medical background, to identify priorities in providing assistance services to the large numbers of urban home-based older adults and thus improve service delivery.

**Supplementary Information:**

The online version contains supplementary material available at 10.1186/s12877-023-04354-7.

## Background

Population aging is one of the most important global trends of the twenty-first century and is thus a primary focus for researchers worldwide [1]. Based on the United Nations Population Fund survey [2], individuals aged ≥ 60 account for 12.3% of the global population, likely rising to nearly 22% by 2050. This trend is especially evident in developed countries, which are rapidly moving toward becoming hyper-aged societies. In contrast, developing countries show a relatively slow movement toward the aging society, although the momentum is increasing [3, 4]. China is classified as a developing nation that possesses the highest concentration of older adults worldwide [5]. Population aging is rapidly increasing for several reasons, such as policies encouraging fertility in the middle of the last century and extending life expectancy [6, 7]. By 2019, people aged > 65 years in China reached approximately 176 million, accounting for 12.6% of the total population [8]. In addition to significantly increasing the social responsibility of caring for older adults, the aging population also poses substantial obstacles to the underdeveloped social service system [9, 10].

Although formal long-term care services for large aging populations have increased significantly, there are still challenges in meeting the increased diversity in the needs of older adults resulting from demographic and socioeconomic changes [11]. Many older adults experience inadequate care or neglect. Furthermore, current care institutions rarely provide extended services, such as rehabilitation support, health maintenance, mental health care, and hospice care [12]. It is thus vital to investigate new and effective forms of care services for older adults to improve the current situation, where many older individuals lack social support for different needs, especially for more urgent needs. In recent years, Chinese researchers have conducted numerous investigations into the emerging forms of older adult care services, such as public welfare social assistance services, home visits by nurses contacted through online appointments, and models for the integration of medical and older adult care, and have put forward multiple strategies to adapt to the national conditions [5, 13, 14].

As a popular community resource for assisting older adults, voluntary social services have always attracted the attention of researchers. Individuals who participate in volunteerism frequently dedicate their time and effort to support the betterment of society and advance social development without seeking compensation or reciprocation [15]. The nature of volunteering can either be formal, planned, and long-term, or spontaneous, informal, and intermittent [16]. Although it is difficult for volunteers to provide daily care for older adults, the use of volunteers has several advantages. For example, a volunteer can support and assist multiple older adults over time. Volunteer teams, led by nursing staff, often have multidisciplinary backgrounds and can provide a variety of services, such as psychological counseling, disease monitoring, and health care [16]. By establishing relationships with their clients while providing practical and effective support, volunteers can positively influence the quality of life and overall well-being of older adults [17]. Therefore, advocating for voluntary services for home-based older adults is important.

The existing research on the application of voluntary services for older adults at home and abroad has focused chiefly on the motivation of the volunteers, their service experience, and the possible impact of voluntary activities [15, 18]. However, there has been little research on older adults’ needs for assistance from voluntary services. A better understanding of these assistance needs in older adults, especially older adults living in urban communities, where they face both difficulties and often disconnection from society [19], can help volunteers understand the potential difficulties faced by older adults, in particular, those associated with advanced age and living alone. This approach will facilitate the identification of groups in more urgent need of help so that appropriate services can be deployed promptly [20, 21]. Additionally, since most older Chinese individuals experienced times of poverty and deprivation, it is often easier to meet their needs in the modern world of abundance and materialism. Hence, it is imperative to identify the potential need for voluntary assistance in older adults in urban communities, specifically within the Chinese culture.

Overall, this study aimed to understand the assistance needs of older adults for voluntary services within urban communities in China and to explore the associated influencing factors. The results of this study can contribute to enhancing the quality of interactions between the small number of volunteers and the large population of older adults, leading to more effective utilization of public welfare resources. Moreover, the conclusions can clarify the actual needs of urban home-based older adults, which will assist in the formulation of hierarchical assistance strategies based on the most urgent needs of older adults.

## Methods

### Study design and participants

A cross-sectional survey was conducted between March 25 and May 15, 2022, using convenience sampling of older individuals from four communities in four Chinese cities: Wuhan, Ezhou, Xinxiang, and Zhengzhou. The inclusion criteria were as follows: (1) Older adults who had lived in Chinese urban communities for more than one year; (2) Older adults who did not receive long-term care services provided by older-adult care institutions; (3) Older adults aged ≥ 60 years; (4) Older adults who were willing to participate in the survey and had provided written informed consent. The exclusion criteria were: (1) Older adults with a history of cognitive impairment or mental illness; (2) Older adults who experienced difficulties in communication due to specific reasons, such as limited hearing or vision, unclear speech, and difficult regional accents. The sample size was selected using the formula for cross-sectional studies [22], $n=\frac{{\mathrm{u}}_{\mathrm{\alpha }/2}^{2}{\upsigma }^{2}}{{\updelta }^{2}}$ Where n is the sample size, a is the significance level, u_0.05/2_ is the area under the corresponding standard normal distribution curve, $\sigma$ is the overall variance, and $\delta$ is the allowable error. We assumed a significance level of 0.05, an area under the corresponding standard normal distribution curve of 1.96, and an acceptable error of 2.50. Pre-tests showed that the $\sigma$ value was 24.44. With an estimate loss of 20% of the sample, a minimum of 459 respondents were required. A total of 560 questionnaires were distributed, of which 516 were ultimately collected, yielding a response rate of 92.1% (516/560). After the exclusion of invalid questionnaires, the effective response rate was 88.9% (498/560). The good response rate may be attributed to the following four points: firstly, prior to the study, our research team conducted multiple surveys in these communities and thus had a good understanding of the situations of many of the older adults, thus facilitating the smooth progress of the research; Secondly, our research group often conducts public welfare activities in these communities and has established good relationships or even friendships with many older adults, thus promoting their cooperation; Thirdly, the support of community leaders and the assistance of staff can also help improve the compliance of older adults; Finally, before conducting the survey, the respondents were contacted by phone in advance. After confirming compliance with the inclusion criteria and informed consent, the use of a face-to-face survey at home greatly improved the effective response rate.

## Instruments

### Questionnaire on demography and other personal characteristics

The questionnaire was self-designed and included information on the following demographic parameters: gender, age, education, religious belief, type of medical insurance, marital status, city level, number of own children, parents, situation, whether living alone or not, willingness to seek care, presence of chronic diseases, receiving a pension, whether receiving financial support from children, and whether receiving financial support from the government. Information on other variables, including willingness to help other older adults, the ability to leave home to handle affairs, and the ability to grasp card payments, were also collected. The specific definitions of these parameters are provided in Appendix I.

### Perceptions of home-based older adults regarding care

Care or caring can be defined as "a nurturing way of relating to a valued other toward whom one feels a personal sense of commitment and responsibility" [23]. The questionnaire was self-designed, and the selection of items was based on a review of domestic and foreign literature, together with an earlier qualitative study we had conducted [24]. The English version of this questionnaire is presented in Appendix II. The questionnaire included two main sections: (1) The first section evaluated the self-care abilities of older adults. Self-care is described as including social support, the addressing of psychological and emotional needs, and the ability to deal with health, illness, or disability [25]. Self-care included 8 items, namely, remaining optimistic, health preservation, self-consolation, seeking treatment, asking for help from others, maintaining interest, showing up for routine physical examinations, and paying attention to self-image. Each item was scored on a 5-point Likert scale from "never = 0" to "frequently = 4"; (2) The second section assessed the level of family and social support experienced by older adults. Family or social care was defined as the care and support provided by the family or society, mainly from the spiritual and psychological aspects [25]. Family care included 4 items, namely, the care received from older adults wives, families of other cohabitants, families of non-cohabitants, and distant relatives. Social care included 7 items: care from government departments, formerly affiliated institutions, old friends, subordinates, students or apprentices, neighbors, visits from medical institutions, and voluntary services. Family and social care-related questions were answered with "yes" or "no" responses. The specific items included in the questionnaire are shown in Appendix III.

### Scale for assessing the needs of home-based older adults for assistance from voluntary services

A self-designed scale was used to assess the assistance needs of older adults in urban communities for voluntary services. The selection of items was based on a review of domestic and foreign literature together with an earlier qualitative study we had conducted [24]. The English version of this scale is presented in Appendix IV. Fifteen experts in older adult-related fields were invited to undertake two rounds of expert consultations, and the I-CVI and S-CVI of the final draft scale were 0.80–1 and 0.96, respectively. Additional exploratory and confirmatory factor analyses were conducted, which demonstrated that the scale exhibited strong reliability and validity. The reliability analysis results indicated that the split-half reliability coefficient was 0.851, while the internal consistency reliability coefficient was 0.969. The validity analysis showed the model's good fitting performance (χ^2^/df = 1.580, NFI = 0.926, CFI = 0.971, RMSEA = 0.053). The scale contained four dimensions, namely, daily life (8 items), health maintenance (7 items), visits and communication (6 items), and social intercourse (6 items), amounting to a total of 27 entries. Each item was scored on a 5-point Likert scale ranging from "absolutely not needed = 1" to "very needed = 5". The total score represented the sum of all items, ranging from 27 to 135 points. A higher score signified an increased need for support from voluntary services, indicating the presence of potentially varied and complex requirements. The Cronbach α coefficient of the scale was 0.968.

### The Activity of Daily Living scale (ADL)

This scale was used to evaluate individual activities of daily living. Activities of daily living comprise basic actions that involve eactivities associated with caring for one's self and body, including personal care, mobility, and eating [26]. The study employed a Chinese version with 10 entries, with a total score of 100 points [27]. The Chinese version of the scale has good validity and reliability. A study test showed that the scale had high concurrent validity, with a Spearman's correlation coefficient of r ≥ 0.92, intraclass correlation coefficient (ICC) of ≥ 0.83, and Cronbach α coefficient of ≥ 0.84 [28]. Higher scores represent better functioning. Therefore, 61 to 99 points indicated mild dependence, 41 to 60 indicated moderate dependence, and ≤ 40 indicated severe dependence. The scale had the advantages of having a relatively simple evaluation, high reliability, and good sensitivity. This scale is also the most widely used and studied evaluation method of activities of daily living at present. In this study, the Cronbach α coefficient of the scale was 0.938.

### Patient Health Questionnaire -9 scale (PHQ-9)

This scale represents a simple and efficient self-assessment tool for depression, based on the American Diagnostic and Statistical Manual of Mental Disorders (DSM-IV) [29]. The present study employed a Chinese version of 9 entries, yielding a total score of 27 points [30]. The Chinese version of the scale has good validity and reliability. A study test showed that the factor load matrix coefficients for all items were > 0.62, the receiver operating characteristic (ROC) curve was ideal, and the Cronbach α coefficient of the scale was 0.89 [31]. On this scale, a higher score represented more severe depression. Therefore, 5 to 9 points indicated mild depression, 10 to 14 points moderate, 15 to 19 points moderate to severe, and 20–27 points indicated severe depression, while a score ≤ 4 indicated no depression. In this study, the Cronbach α coefficient of the scale was 0.928.

### Data collection

Before the investigation, the investigators contacted and sought the consent of community leaders or their departments in the four communities to ensure the smooth progress of the investigation. Subsequently, all investigators received unified training to clarify the purpose and precautions of the study, after which the investigators obtained contact and residential information for older adult residents from the community management departments. To preserve the confidentiality of personal information, the investigators contacted older adults who met the inclusion criteria or their families one by one via telephone within the confines of the community management department's office area.

After confirming that the participants conformed to the inclusion and exclusion criteria and obtaining their informed consent, community workers accompanied the investigators to the participants' homes. Before distributing the questionnaire, the investigators again confirmed whether the prospective participants conformed with the exclusion criteria and the participants then signed informed consent forms. The investigators read each item of the questionnaire neutrally and impartially, as all participants were over 60 years old and many had relatively low levels of education. If conditions permitted, participants could also complete the paper questionnaire independently or use the WeChat app to scan QR codes and complete online questionnaires. The investigators supervised and provided necessary explanations during the filling in of the questionnaire to ensure the quality of the questionnaire. After completion of the survey, the investigators assessed whether the questionnaires had been properly completed. In the case of questionnaires with quality issues, such as incompleteness, the investigators returned them to the participants for revision. The investigation followed the principles of anonymity and confidentiality.

### Data analysis

After the survey, questionnaires with errors and poor quality were excluded. All analyses were performed using SPSS 19.0 statistical software. The age and total score of the assistance needs for voluntary services were tested via the K-S test and evaluated with Q-Q diagrams. The results revealed that age was non-normally distributed, while the total score of the need for assistance from voluntary services showed an approximately normal distribution. Percentages (%) and medians (interquartile range [IQR]) were used for the descriptions of the demographic characteristics and perceived needs for assistance of the home-based older adults. Means (SD) were used for the presentation of the total score of the need for assistance from voluntary services. One-way analysis of variance or independent-sample t-tests were used to identify significant variables related to the need for assistance from voluntary services. The dependent variable represented the need for support from voluntary services among older persons. Significant influencing factors identified in the single-factor analysis were used as independent variables in a multivariate linear (stepwise) regression analysis. The significance level was set at 0.05.

## Results

### Demographic and personal characteristics of the participants

The mean age of the participants was 72 years (IQR: 66–80 years). Over half of the subjects (58.8%) were female, and most exhibited chronic disease complications (74.9%). Overall, 35.7% of the participants were educated to high school level or above, 90.4% had religious beliefs, and 71.1% had urban medical insurance. Most participants were married (68.5%), had no caregivers (67.7%), and 51.6% lived in megacities. Only 17.5% of the participants lived alone, and most were willing to help other older adults (72.5%). Most participants were married (68.5%), had no caregivers (67.7%), and 51.6% lived in megacities. Most participants had 2–3 children (74.1%) and no living parents (82.0%). Although the majority of participants received a pension (75.7%), few received financial support from their children (36.1%) or the government (10.6%). Most participants felt comfortable going out to handle affairs (76.5%) but 92.8% had not grasped the use of card payments.

### Participants’ assistance needs for voluntary services

The mean score of the need for assistance from voluntary services was 88.60 (SD = 24.37) (scale median: 81). The average scores for each dimension ranging from high to low were health maintenance (M = 3.64, SD = 1.08), visits and communication (M = 3.49, SD = 1.04), social intercourse (M = 3.33, SD = 1.08), and daily life (M = 2.78, SD = 1.08) (Table 1). Regarding the average scores for individual entries in the older adults' assistance needs for voluntary services, it was found that the top five scores were for "Health care and disease prevention guidance" (M = 3.74, SD = 1.15), "A timely response when asking for help" (M = 3.71, SD = 1.15), "Medication instruction" (M = 3.69, SD = 1.19), "Blood pressure measurement, blood glucose monitoring, and other physical examinations" (M = 3.66, SD = 1.23), and "Being respected and appreciated by visiting volunteers" (M = 3.64, SD = 1.11). The comprehensive results are presented in Appendix V.

**Table 1:** The scores for four dimensions of assistance needs for voluntary social services (*N* = 498)

Variables	Entries	Score ranges	Mean(SD)	Entries mean(SD)
Assistance needs for social voluntary services	27	27–135	88.60 (24.37)	3.28 (0.90)
Daily life	8	8–40	22.20 (8.67)	2.78 (1.08)
Health maintenance	7	7–35	25.46 (7.59)	3.64 (1.08)
Visits and communication	6	6–30	20.95 (6.23)	3.49 (1.04)
Social intercourse	6	6–30	19.98 (6.46)	3.33 (1.08)

SD indicates standard deviation

### Factors influencing assistance needs for voluntary services

Tables 2 and 3 demonstrate the differences in the assistance needs for voluntary services, based on the characteristics of home-dwelling older adults in Chinese cities and the experience of being cared for in the past, respectively. The results indicated that the purchase of the new rural cooperative medical insurance, living in a city of average size rather than a megacity, non-single children, presence of one or both living parents, being taken care of by others, willingness to help other older adults, lack of a pension, lack of financial support from children, inability to venture out to handle affairs, or inability to perform card payments were associated with an elevated need for assistance from voluntary services. In contrast, not paying attention to health preservation, having an ability to self-comfort, seeking treatment, asking for help from others, obtaining social care from government departments, subordinates, students or apprentices, neighbors, visited medical institutions and voluntary social services were associated with reduced assistance needs for voluntary services Further S–N-K analysis revealed that the scores of assistance needs for voluntary services were lower in older adults with one child, those whose parents were no longer living, who were not concerned about preserving their health, had the ability to self-comfort, sought treatment, and asked for help from others. Alternately, the scores of older adults who had obtained the new rural cooperative medical insurance were higher than other groups.

**Table 2:** Univariate analysis of demographic data of the home-based elderly (*N* = 498)

Variables	Mean (SD)	*N(%)*	*t/F*	*P*
**Gender**			0.666 ^a^	0.450
Male	87.73(24.90)	205(41.2)		
Female	89.20(24.01)	293(58.8)		
**Age**			1.180 ^b^	0.308
60–69	89.82(23.22)	202(40.6)		
70–79	89.25(23.11)	170(34.1)		
80 or above	85.75(27.58)	126(25.3)		
**Education**			0.791 ^b^	0.454
Primary school education or under	90.33(21.61)	187(37.6)		
Junior high school education	87.96(24.64)	133(26.7)		
Senior high school education or above	87.25(26.80)	178(35.7)		
**Religious belief**			1.858 ^a^	0.064
Yes	94.79(21.11)	48(9.6)		
No	87.94(24.62)	450(90.4)		
**Type of medical insurance**			9.920 ^b^	0.000*
Urban medical insurance	86.69(24.83)	354(71.1)		
New rural cooperative medical insurance	96.91(17.65)	113(22.7)		
Other insurance	80.03(32.20)	31(6.2)		
**Marital status**			-0.782 ^a^	0.435
Single	87.34(23.61)	157(31.5)		
Married	89.18(24.72)	341(68.5)		
**City level**			-4.710 ^a^	0.000*
Megacity	83.72(25.80)	257(51.6)		
General city	93.80(21.61)	241(48.4)		
**Number of own children**			4.359 ^b^	0.005*
0	94.00(22.29)	7(1.4)		
1	82.02(26.57)	129(25.9)		
2	90.82(23.83)	188(37.8)		
≥ 3	90.86(22.54)	174(34.9)		
**Parents’ situation**			3.572 ^b^	**0.029**
All alive	95.18(21.94)	45(9.0)		
A single parent died	94.36(24.73)	45(9.0)		
Both died	87.24(24.42)	408(82.0)		
**Living alone**			-0.512 ^a^	0.609
Yes	88.85(24.30)	87(17.5)		
No	87.38(24.79)	411(82.5)		
**Whether taken care of**			-3.280 ^a^	0.001*
Yes	93.73(24.02)	125(32.3)		
No	86.15(24.18)	337(67.7)		
**Whether suffering from chronic diseases**			0.931 ^a^	0.352
Yes	88.01(24.59)	373(74.9)		
No	90.35(23.70)	125(25.1)		
**Whether willing to help other elderly people**			5.504 ^a^	0.000*
Yes	92.19(21.65)	361(72.5)		
No	79.12(28.35)	137(27.5)		
**Whether receiving a pension**			-2.740 ^a^	0.006*
Yes	86.91(25.46)	377(75.7)		
No	93.84(19.77)	121(24.3)		
**Whether receiving financial support from children**			3.474 ^a^	0.001*
Yes	85.77(26.36)	180(36.1)		
No	93.58(19.47)	318(63.9)		
**Whether receiving financial support from government**			-1.791 ^a^	0.074
Yes	82.94(29.50)	53(10.6)		
No	89.27(23.63)	445(89.4)		
**Whether feeling comfortable going out to handle affairs**			-3.260 ^a^	0.001*
Yes	86.64(25.22)	381(76.5)		
No	94.96(20.15)	117(23.5)		
**Whether can grasp card payments**			-2.640 ^a^	0.009*
Yes	78.33(23.22)	36(7.2)		
No	89.40(24.29)	462(92.8)		

SD indicates standard deviation, t indicates t-test; F indicates F-test, *P* indicates significance level, ^a^ indicates the independent-sample t test, ^b^ indicates one-way analysis of variance; Bold font indicates statistically significant differences (*P* < 0.05), * indicates statistically significant difference (*P* < 0.01)

**Table 3:** Univariate analysis of past care experience of the home-based elderly (*N* = 498)

Dimensions	Variables	Mean (SD)					*t/F*	*P*
		**Never**	**Occasionally**	**Sometimes**	**Often**	**Frequently**		
		**No**	**Yes**					
Self-care	Maintaining optimism	92.75(9.67)	89.00(20.89)	91.35(23.10)	87.96(22.20)	87.84(27.80)	0.388 ^b^	0.817
	Paying attention to health preservation	78.15(27.07)	85.45(21.25)	90.94(21.98)	87.35(22.65)	92.00(27.76)	2.508 ^b^	**0.041**
	Self-comfort	69.70(18.95)	88.41(22.86)	92.36(20.53)	86.32(22.60)	90.28(28.99)	2.787 ^b^	**0.026**
	Seeking treatment	65.17(20.65)	78.20(20.27)	88.98(21.72)	90.98(20.37)	89.38(29.82)	3.961 ^b^	0.004*
	Asking for help from others	75.00(26.19)	82.61(23.38)	89.18(21.94)	91.25(21.09)	92.42(29.67)	4.836 ^b^	0.001*
	Keeping interest cultivation	83.32(22.70)	84.51(24.26)	91.78(21.23)	88.83(21.19)	89.65(29.53)	1.490 ^b^	0.204
	Keeping physical examination	82.33(29.16)	91.12(20.98)	90.80(20.15)	87.65(21.80)	87.78(31.65)	1.259 ^b^	0.285
	Paying attention to self-image	87.45(26.72)	85.52(23.60)	89.90(20.61)	87.39(22.75)	93.62(30.62)	1.326 ^b^	0.259
Family care	From elderly wives	88.98(24.80)	87.85(23.57)				0.490 ^b^	0.624
	From families of other cohabitants	89.18(24.13)	88.14(24.58)				0.474 ^b^	0.636
	From families of non-cohabitants	88.69(24.30)	87.30(25.56)				0.315 ^b^	0.753
	From distant relatives	89.10(23.90)	86.59(26.18)				0.921 ^b^	0.358
Social care	From government departments	90.58(23.63)	84.74(25.37)				2.546 ^b^	**0.011**
	From former affiliated institutions	88.59(25.14)	88.60(24.01)				0.003 ^b^	0.997
	From old friends	89.69(24.35)	86.20(24.30)				1.485 ^b^	0.138
	From subordinates, students or apprentices	92.16(23.72)	87.29(24.50)				1.984 ^b^	**0.048**
	From neighbors	90.04(23.44)	84.29(26.58)				2.294 ^b^	**0.022**
	From visited medical institutions	92.46(22.67)	85.80(25.19)				3.037 ^b^	0.003*
	From social voluntary services	96.11(23.52)	86.00(24.14)				4.112 ^b^	0.000*

SD indicates standard deviation, t indicates t-test; F indicates F-test, *P* indicates significance level, ^a^ indicates independent-sample t-test, ^b^ indicates one-way analysis of variance, Bold font indicates statistically significant differences (*P* < 0.05), * indicates statistically significant difference (*P* < 0.01)

The results of the bivariate correlation analysis revealed a statistically weak negative correlation between the scores of assistance needs for voluntary services and activities of daily living (*r* = **-**0.106, *P* = 0.018). Furthermore, there was a statistically significant positive correlation between the scores of assistance needs for voluntary services and depression (*r* = 0.446, *P* < 0.001).

### Multivariate linear regression analysis of the factors influencing the need for assistance from voluntary services

We then used multivariate linear regression analysis to identify significant variables associated with the need for assistance from voluntary services in Chinese city-dwelling older adults. Based on t-test, ANOVA, and Pearson's correlation analyses, statistically significant variables (*P* < 0.05) were entered into the regression model, demonstrating that the scores for depression, willingness to help other older adults, paying close attention to health preservation, number of living children, asking for help from others, social care from visited medical institutions, and the ability to self-comfort were critical predictors of the need for assistance from voluntary services. The results of the multivariate linear regression analysis are summarized in Table 4.

**Table 4:** Multivariate linear regression analysis of the factors influencing assistance needs for social voluntary services among the home-based elderly (*N* = 498)

Variables	*B*	*SE*	*β*	*t*	*P*	*95% CI for B*
Constant	92.664	4.259		21.757	0.000	84.296 ~ 101.033
The score of depression	1.954	0.173	0.440	11.299	0.000	1.614 ~ 2.294
Whether to be willing to help other elderly people	-8.882	2.126	-0.163	-4.178	0.000	-13.058 ~ -4.705
Paying attention to health preservation = frequently	7.314	2.098	0.136	3.486	0.001	3.191 ~ 11.436
Number of own children = 1	-5.366	2.132	-0.097	-2.517	0.012	-9.555 ~ -1.178
Asking for help from others = occasionally	-6.250	2.572	-0.093	-2.430	0.015	-11.303 ~ -1.196
Self-comforting = never	-16.289	6.653	-0.094	-2.448	0.015	-29.360 ~ -3.218
Social care from visited medical institutions	-4.402	1.935	-0.089	-2.275	0.023	-8.205 ~ -0.600

B indicates non-standardized regression coefficient; SE indicates standard error, β indicates regression coefficient, t indicates t-test, P indicates significant level, 95% CI for B indicates 95% confidence interval. R^2^ = 0.295, adjusted R^2^ = 0.285, *F* = 29.262, *P* < 0.001

## Discussion

This study aimed to explore the need for assistance from voluntary services among home-based older adults in Chinese cities and to identify the influencing factors. Unlike previous studies [32, 33], we obtained data on the care needs of older adults living at home from the perspective of public welfare care services. Research suggests that individuals who receive significant levels of social support have better health and well-being, improved physical wellness, less depression, more life satisfaction, and reduced loneliness [34]. These elements play a crucial role in healthy aging. As volunteers are often unable to provide day-care services and thus cannot offer continuous and meticulous care, it is more practical to provide a wide range of support services to older adults, such as psychological guidance, health maintenance, and interest cultivation, which are quite different from the routine care services provided by home care workers. An understanding of the specific needs of home-based older adults for assistance from voluntary services is highly beneficial for social volunteers who wish to deliver more purposeful and effective aid that pertains to the actual life needs of older adults. In addition, considering that older adults often require healthcare services [35], it is equally important to clarify their needs for medical services.

In this study, we demonstrated that the overall need for assistance from voluntary services among home-based older adults in Chinese cities was far above the moderate level, clearly higher than expected. The results also showed that although the majority of older adults included in the study were young-old (i.e., less than 80, accounting for 74.7% of the overall study population), with lower daily life needs, they showed greater demands for medical services, interpersonal communication, and other broad high-level assistance, indicating that with the development of social economy, the demands of home-based older adults in Chinese cities have not only increased but have also become more diverse and complex. These findings are consistent with the findings of Zhou et al. [5] who observed an unexpected increase in the demands of older individuals for medical services. As a result, it was advised to publicize the idea of healthy aging. A healthy lifestyle is crucial to promoting healthy aging and should include frequent health examinations, good nutrition, care of mental health, and adequate social engagement [36]. Evidence indicates that such interventions can contribute to both the length and quality of life, thereby significantly reducing the overall demand for healthcare among older adults [36, 37].

We also assessed the desires of urban home-based older adults to interact with the external world, involving both passive visits and active venturing out of the home to meet with others. The results indicated that compared to daily life, older adults had stronger overall demands in these categories. A study in Ethiopia also described the tendency of older adults to "prefer greeting rather than eating" [38]. Some studies even revealed that long-term social isolation leads to unfulfilled needs for social communication, seriously affecting the quality of life of older adults, and also increasing the risk of depression [39–41].

The most prominent specific needs of older individuals living at home in cities were highlighted through further study of the results. In terms of health maintenance, the demand for health care and guidance in disease prevention, medication instruction, physical examinations, and the need for receiving a timely response when seeking help were much higher than other needs. Interestingly, we discovered that older adults not only hoped to receive specific instructions on health maintenance and disease prevention but also wished to establish close contact with volunteers, led by medical staff, to obtain professional advice and even aid when necessary or in an emergency. A previous study supported this finding that social networks can increase opportunities and help vulnerable older adults access the benefits of volunteer services [42]. Another previous study revealed that older adults often struggle to meet their medical needs due to inconvenient transportation, difficulty keeping appointments, and other reasons [43, 44]. Even worse, if not supported, some older adults may fall in their homes and not be found for a long time, with potential life-threatening consequences [45, 46]. Hence, it is necessary to encourage volunteers, particularly those with a medical background, to establish close contact with older adults living at home in urban environments, even those with no current medical needs, to provide assistance when necessary or during an emergency. Another notable special requirement pertained to social interaction. Typically, visiting volunteers should offer an adequate level of respect and appreciation. This result was consistent with a previous study that demonstrated that 73% of the rural community-dwelling older adults agreed that respect is the most desired trait amongst volunteers [47]. Perceived respect and appreciation from younger generations is proven to be related to goal setting and psychological well-being in older adults [48]. This suggests that volunteers should show sincere caring behavior when meeting the emotional needs of urban home-based older adults.

The results of the study also showed that the scores of depression, willingness to help other older adults, seeking help from others, paying close attention to maintaining health, the ability to self-comfort, receiving social care from visited medical institutions, and the number of children were independent factors influencing the need for assistance from voluntary services in urban home-based older adults. Older adults with higher levels of depression generally have greater assistance needs, due to elevated perceptions of physical, psychological, or social crises. Hence, these people may prefer someone to check in on them, chat, accompany them to conduct outside affairs, or provide assistance [49, 50]. It has been reported that depressive symptoms in older adults may be correlated with dementia, and even suicidal ideation [51, 52]. However, the rate at which older adults at risk of depression who visit outpatient departments is very low, and it is thus difficult for them to receive timely intervention [53]. Fortunately, home visits by volunteers can significantly reduce psychological distress and even suicidal ideation among older adults, especially women [54, 55]. Therefore, volunteers, especially those with medical backgrounds, should be encouraged to provide appropriate help and psychological support to older adults with depressive tendencies to prevent the aggravation of depressive symptoms.

Furthermore, we observed that older adults who tended to seek assistance from others had higher assistance needs and were often better at expressing themselves and their specific requests for assistance. Interestingly, the study also found that older adults who were proactive in their intention to help other older adults also had higher assistance needs. It is sometimes the case that older adults who are helpful to other older adults have higher unmet needs of their own that go unnoticed. The important discovery of a direct relationship between willingness to help other older adults and higher assistance requirements is enlightening in terms of which demographic requires the greatest care. Thus, we must not ignore the assistance needs of older adults who are helpful to others. On the contrary, these people may need more help. Thus, providing help to these older adults may necessitate better targeting, which may indirectly benefit other older adults. Furthermore, mutual aid among older adults may benefit both sides [56]. For example, a study found that older adults living in areas with high levels of volunteer group participation, irrespective of whether they participated in volunteer groups, had a lower risk of developing depressive symptoms [57].

The study also revealed that older adults who paid frequent attention to health preservation, including eating a balanced diet and exercising, had higher assistance needs. This was presumably because older persons focused on these specifics tended to pursue a higher quality of life, making them more willing to report unmet demands. According to Cohen-Mansfield and Frank [20], older individuals who value healthy lifestyle habits may seek more services and be more willing to accept assistance and support. The results also showed that older adults lacking the ability to self-comfort had lower needs for assistance. Likewise, a positive mindset leads to a more optimistic attitude toward life [58], and these older adults are more inclined to seek family or social support to address their needs. These crucial discoveries revealed that there should be mutual understanding and cooperation between older adults and volunteers rather than a one-sided concentration of effort. If older adults have a variety of unmet needs but are unwilling to express them, no matter how professional and active volunteers are, they cannot assist. Therefore, before the provision of volunteer services, the government and even society should vigorously encourage older adults who neglect self-care to develop healthy lifestyles [34]. This would help improve their positive attitudes toward and compliance with volunteer services, as well as contribute to their own healthy aging [36].

Additionally, we demonstrated that receiving better social care from visited medical institutions was beneficial for addressing unmet needs for assistance in older adults. This result is consistent with previous studies showing that older adults who received visiting nursing services were less likely to report unmet needs [59, 60]. An increasing body of evidence shows that professionally trained volunteers who provide home services to individuals with chronic diseases improve both the quality of life and family well-being of older adults [61]. Therefore, society should encourage volunteers with medical backgrounds to actively participate in providing assistance services to older adults at home to meet their diverse needs and as a necessary supplement to continuing care after hospital discharge.

Another surprising finding was that in addition to the elevated demands for assistance shown by older adults without children, the same applied to older adults with children. This finding was contrary to an extensive body of literature that suggested that more children are associated with greater family support [62–65]. Although it is often assumed that the presence of more children will be able meet the needs of older adults [64], this is not always the case. Suitor et al. [66] suggested that perceptions of parental favoritism by offspring may exacerbate tensions among various family members and thus may indirectly affect the quality of care provided to parents. Another study indicated that the quality of care provided to older adults depends more on the resourcefulness of the children rather than the number of children [65].

Therefore, volunteers should not only focus on older adults without children but also should not ignore the assistance needs of older adults with many children. This would necessitate a comprehensive assessment of the ability of the children to provide care. Meanwhile, volunteers should not neglect encouraging the children to provide care for their parents, which would significantly reduce their feelings of loneliness and improve their level of happiness [34]. In addition, in contrast to previous studies [33, 67, 68], several crucial variables associated with group differences in the need for assistance from voluntary services, such as living alone and difficulties with daily living activities, were not entered into the final regression equation as they did not reach significance in the present study. These variables may be affected by confounding factors. As such, the importance of these variables in volunteer assistance service requires further examination.

### Limitations

This study has several limitations. First, the main scale used in the study was self-designed. Although the process was strictly controlled, the design may not have been as mature as others. Therefore, the scale requires further improvement and supplementation. Second, convenience sampling limits the generalizability of the present findings to the overall population of older adults. Third, there may have been unintentional regional biases since this was a multi-center study. Fourth, all the questionnaires in the survey were self-reporting scales, which may have led to some bias in the results. Fifth, the study was cross-sectional and did not provide data on temporal relationships and cause-effect inferences between the variables, thus the associations should be taken with caution. Additionally, the study was primarily done in central China, which may not adequately represent the country as a whole. Hence, this investigation needs to be repeated with more data from all over China. Finally, the sample was age-skewed in that there were, overall, fewer oldest-old (i.e., 80 or above, accounting for 25.3% of the entire study population) because older adults who agreed to be interviewed and were able to express their needs adequately were generally of the younger-old category.

## Conclusions

In Chinese urban regions, older adults living at home were found to have above-moderate assistance needs for voluntary services. Compared to daily care and life assistance, these older adults exhibited greater needs in terms of health maintenance, social intercourse, and the wish for visits and communication, particularly in the specific areas of health care and guidance in disease prevention, medication instruction, physical examinations, obtaining respect and appreciation from visiting volunteers, and obtaining timely responses when asking for help. Older adults living in Chinese urban communities who experienced a higher level of depression were found to be more willing to help other older adults, obtain no social care from visited medical institutions, attach greater importance to health preservation, can self-comfort, wish to seek help from others, and have either more children or no children, and thus reported greater needs for assistance from voluntary services. These findings will be helpful to social volunteers, especially those with a medical background. This work clarified the priority of providing assistance services to urban home-based older adults and suggested improvements in their services.

### Supplementary Information

**Additional file 1:** **Appendix I.** Definition and measurement of each demographic characteristics. **Appendix II.** English version of the questionnaire on perceptions of home-based older adults regarding care. **Appendix III.** Specific items of perceptions of the home-based older adults regarding care questionnaire. **Appendix IV.** English version of the scale for assessing the needs of home-based older adults for assistance from voluntary services. **Appendix V.** The average score of all entries in the scale for assessing the needs of the home-based elderly for assistance from voluntary services.

## Data Availability

The datasets generated and analyzed during the study are available from the corresponding author by reasonable request.
