# Supplementary material for: Factors associated with the perceived need for assistance from voluntary services in home-based older adults in Chinese urban areas: a cross-sectional study
Source: BMC Geriatr. 2023 Oct 6;23:624. doi: 10.1186/s12877-023-04354-7 (PMC10557159; doi:10.1186/s12877-023-04354-7)
Supplement: Supplementary file 1 — Additional file 1: Appendix I. Definition and measurement of each demographic characteristics. Appendix II. English version of the questionnaire on perceptions of home-based older adults regarding care. Appendix III. Specific items of perceptions of the home-based older adults regarding care questionnaire. Appendix IV. English version of the scale for assessing the needs of home-based older adults for assistance from voluntary services. Appendix V. The average score of all entries in the scale for assessing the needs of the home-based elderly for assistance from voluntary services. [file 12877_2023_4354_MOESM1_ESM.docx]

Appendix I.

Definition and measurement of each demographic characteristics.

| **Variables** | **The definition** |
| --- | --- |
| **Gender** |  |
| Male |  |
| Female |  |
| **Age** |  |
| 60-69 |  |
| 70-79 |  |
| 80 or above |  |
| **Education** |  |
| Primary school education or under |  |
| Junior high school education |  |
| Senior high school education or above |  |
| **Religious belief** | Defined as any religious belief |
| Yes |  |
| No |  |
| **Type of medical insurance** |  |
| Urban medical insurance | Divided according to the source of medical insurance |
| New rural cooperative medical insurance |  |
| Other insurance |  |
| **Marital status** |  |
| Single/ Bachelor | Including unmarried, divorced, and widowed |
| Married |  |
| **City level** |  |
| Megacity | Defined as a large city with a population of over 10 million |
| General city | Defined as a city with a population of no more than 10 million |
| **Number of own children** |  |
| 0 |  |
| 1 |  |
| 2 |  |
| ≥3 |  |
| **Parents’ situation** |  |
| All alive |  |
| A single parent died |  |
| Both died |  |
| **Living alone** |  |
| Yes |  |
| No |  |
| **Whether taken care of** | Defined as whether to receive care from anyone else |
| Yes |  |
| No |  |
| **Whether suffering from chronic diseases** | Defined as having one or more chronic diseases |
| Yes |  |
| No |  |
| **Whether willing to help other older adults** | Defined as having the willingness to help other older adults |
| Yes |  |
| No |  |
| **Whether receiving a pension** | Defined as whether to receive monthly pension payments from the original affiliated institution or government department |
| Yes |  |
| No |  |
| **Whether receiving financial support from children** | Defined as whether to receive monthly subsidies from children |
| Yes |  |
| No |  |
| **Whether receiving financial support from government** | Defined as whether to receive subsidies from government department |
| Yes |  |
| No |  |
| **Whether being able to go out to handle affairs** | Defined as going out alone to handle affairs without the help of others |
| Yes |  |
| No |  |
| **Whether grasping card payments** | Defined as using a savings or credit card for payment without assistance from others |
| Yes |  |
| No |  |

Appendix II.

English version of the questionnaire on perceptions of home-based older adults regarding care.

| Q1 | I strive to maintain a positive and optimistic attitude. |
| --- | --- |
| Q2 | I focus on health preservation to improve my health, such as exercise and a healthy diet. |
| Q3 | When I encounter setbacks and low emotions, I will comfort and enlighten myself. |
| Q4 | When I am sick, I actively seek treatment and recovery. |
| Q5 | When I encounter difficulties or feel helpless, I actively seek help from people around me. |
| Q6 | I am passionate about cultivating or adhering to my interests and hobbies. |
| Q7 | I will undergo a health check up. |
| Q8 | I pay attention to dressing up and giving myself confidence in life. |
| Q9 | I have received care from elderly wives. |
| Q10 | I have received care from families of other cohabitants. |
| Q11 | I have received care from families of non-cohabitants. |
| Q12 | I have received care from distant relatives. |
| Q13 | I have received care from government departments. |
| Q14 | I have received care from former work units. |
| Q15 | I have received care from old friends. |
| Q16 | I have received care from subordinates, students or apprentices. |
| Q17 | I have received care from neighbors. |
| Q18 | I have received care from visited medical institutions. |
| Q19 | I have received care from social voluntary services. |

Appendix III.

Specific items of perceptions of the home-based older adults regarding care questionnaire.

| **Dimensions** | **Variables** | **Specific items** |
| --- | --- | --- |
| Self-care | Maintaining optimism | I strive to maintain a positive and optimistic attitude. |
|  | Paying attention to health preservation | I focus on health preservation to improve my health, such as exercise and a healthy diet. |
|  | Self-comfort | When I encounter setbacks and low emotions, I will comfort and enlighten myself. |
|  | Seeking treatment | When I am sick, I actively seek treatment and recovery. |
|  | Asking for help from others | When I encounter difficulties or feel helpless, I actively seek help from people around me. |
|  | Keeping interest cultivation | I am passionate about cultivating or adhering to my interests and hobbies. |
|  | Keeping physical examination | I will undergo a health check up. |
|  | Paying attention to self-image | I pay attention to dressing up and giving myself confidence in life. |
| Family care | From elderly wives | I have received care from elderly wives. |
|  | From families of other cohabitants | I have received care from families of other cohabitants. |
|  | From families of non-cohabitants | I have received care from families of non-cohabitants. |
|  | From distant relatives | I have received care from distant relatives. |
| Social care | From government departments | I have received care from government departments. |
|  | From former affiliated institutions | I have received care from former work units. |
|  | From old friends | I have received care from old friends. |
|  | From subordinates, students or apprentices | I have received care from subordinates, students or apprentices. |
|  | From neighbors | I have received care from neighbors. |
|  | From visited medical institutions | I have received care from visited medical institutions. |
|  | From social voluntary services | I have received care from social voluntary services. |

Appendix IV.

English version of the scale for assessing the needs of home-based older adults for assistance from voluntary services.

| Q1 | Obtain assistance in purchasing groceries or shopping from voluntary services. |
| --- | --- |
| Q2 | Obtain assistance in delivering meals or cooking from voluntary services. |
| Q3 | Obtain assistance in maintaining personal hygiene from voluntary services. |
| Q4 | Obtain assistance in activities within the room from voluntary services. |
| Q5 | Obtain assistance in going out for activities or walks from voluntary services. |
| Q6 | Obtain assistance in cleaning from voluntary services. |
| Q7 | Obtain guidance on how to use mobile phones or the internet from voluntary services. |
| Q8 | Obtain assistance in handling deposits, withdrawals, or wealth management at the bank from voluntary services. |
| Q9 | Obtain assistance in measuring blood pressure, blood sugar, or conducting other physical examinations from voluntary services. |
| Q10 | Obtain medication instruction from voluntary services. |
| Q11 | Obtain guidance on health care and disease prevention from voluntary services. |
| Q12 | Obtain guidance on fall prevention from voluntary services. |
| Q13 | Obtain accompany for medical treatment or physical examination from voluntary services. |
| Q14 | Obtain timely response when seeking sought for help from voluntary services. |
| Q15 | Obtain guidance on physical rehabilitation and functional exercise from voluntary services. |
| Q16 | Be accompanied in chatting by volunteers. |
| Q17 | Obtain encouragement on speaking up and respond from voluntary services. |
| Q18 | Be celebrated for birthdays or holidays by volunteers. |
| Q19 | Be identified the negative emotions and obtain suggestions by volunteers. |
| Q20 | Be respected and appreciated by volunteers. |
| Q21 | Obtain strategies to address the concerns about the families from voluntary services. |
| Q22 | Obtain assistance in registering or contacting for social activities from voluntary services. |
| Q23 | Obtain assistance in participate in social activities from voluntary services. |
| Q24 | Obtain assistance in organize social activities from voluntary services. |
| Q25 | Obtain assistance in meeting more friends, clubs, or organizations from voluntary services. |
| Q26 | Be accompanied by volunteers in doing things they are interested in. |
| Q27 | Obtain a platform to showcase or cultivate one's interests and hobbies from voluntary services. |

The subject of all questions is 'home-based older adults'.

Appendix V.

The average score of all entries in the scale for assessing the needs of the home-based elderly for assistance from voluntary services.

| **Dimensions** | **Iterms** | **Minimum** | **Maximum** | **Mean (SD)** |
| --- | --- | --- | --- | --- |
| Life  assistance | 1. Obtain assistance in purchasing groceries or shopping | 1 | 5 | 2.81 (1.29) |
|  | 1. Obtain assistance in delivering meals or cooking | 1 | 5 | 2.87 (1.30) |
|  | 1. Obtain assistance in maintaining personal hygiene | 1 | 5 | 2.37 (1.32) |
|  | 1. Obtain assistance in activities within the room | 1 | 5 | 2.34 (1.28) |
|  | 1. Obtain assistance in going out for activities or walks | 1 | 5 | 2.80 (1.31) |
|  | 1. Obtain assistance in cleaning | 1 | 5 | 3.04 (1.33) |
|  | 1. Obtain guidance on how to use mobile phones or the internet | 1 | 5 | 3.19 (1.30) |
|  | 1. Obtain assistance in handling deposits, withdrawals, or wealth management at the bank | 1 | 5 | 2.79 (1.33) |
| Health  maintenance | 1. Obtain assistance in measuring blood pressure, blood sugar, or conducting other physical examinations | 1 | 5 | 3.66 (1.23) |
|  | 1. Obtain medication instruction | 1 | 5 | 3.69 (1.19) |
|  | 1. Obtain guidance on health care and disease prevention | 1 | 5 | 3.74 (1.15) |
|  | 1. Obtain guidance on fall prevention | 1 | 5 | 3.64 (1.19) |
|  | 1. Obtain accompany for medical treatment or physical examination | 1 | 5 | 3.50 (1.25) |
|  | 1. Obtain timely response when seeking sought for help | 1 | 5 | 3.71 (1.15) |
|  | 1. Obtain guidance on physical rehabilitation and functional exercise | 1 | 5 | 3.52 (1.24) |
| Visits and  communication | 1. Be accompanied in chatting | 1 | 5 | 3.47 (1.17) |
|  | 1. Obtain encouragement on speaking up and respond | 1 | 5 | 3.46 (1.18) |
|  | 1. Be celebrated for birthdays or holidays | 1 | 5 | 3.33 (1.18) |
|  | 1. Be identified the negative emotions and obtain suggestions | 1 | 5 | 3.52 (1.15) |
|  | 1. Be respected and appreciated | 1 | 5 | 3.64 (1.11) |
|  | 1. Obtain strategies to address the concerns about the families | 1 | 5 | 3.54 (1.19) |
| Social  intercourse | 1. Obtain assistance in registering or contacting for social activities | 1 | 5 | 3.23 (1.17) |
|  | 1. Obtain assistance in participate in social activities | 1 | 5 | 3.27 (1.18) |
|  | 1. Obtain assistance in organize social activities | 1 | 5 | 3.42 (1.16) |
|  | 1. Obtain assistance in meeting more friends, clubs, or organizations | 1 | 5 | 3.33 (1.16) |
|  | 1. Be accompanied in doing things they are interested in | 1 | 5 | 3.37 (1.20) |
|  | 1. Obtain a platform to showcase or cultivate one's interests and hobbies | 1 | 5 | 3.37 (1.15) |
